# Adoptive transfer of tumor-infiltrating lymphocytes in melanoma: a viable treatment option

**DOI:** 10.1186/s40425-018-0391-1

**Published:** 2018-10-03

**Authors:** Maartje W. Rohaan, Joost H. van den Berg, Pia Kvistborg, John B. A. G. Haanen

**Affiliations:** 1 Department of Medical Oncology, The Netherlands Cancer Institute (NKI), Plesmanlaan 121, 1066 CX Amsterdam, The Netherlands; 2 Biotherapeutics Unit, The Netherlands Cancer Institute, Plesmanlaan 121, 1066 CX Amsterdam, The Netherlands; 3 Division of Molecular Oncology and Immunology, The Netherlands Cancer Institute, Plesmanlaan 121, 1066 CX Amsterdam, The Netherlands

**Keywords:** Melanoma, Adoptive cell therapy, Tumor-infiltrating lymphocytes, Immunotherapy, Lymphodepletion, Interleukin-2, Antigen recognition, Combination therapy

## Abstract

The treatment of metastatic melanoma patients with autologous tumor-infiltrating lymphocytes (TIL) shows robust, reproducible, clinical responses in clinical trials executed in several specialized centers over the world. Even in the era of targeted therapy and immune checkpoint inhibition, TIL therapy can be an additional and clinically relevant treatment line. This review provides an overview of the clinical experiences with TIL therapy thus far, including lymphodepleting regimens, the use of interleukin-2 (IL-2) and the associated toxicity. Characteristics of the TIL products and the antigen recognition pattern will be discussed, as well as the current and upcoming production strategies, including the selective expansion of specific fractions from the cell product. In addition, the future potential of TIL therapy in melanoma and other tumor types will be covered.

**Electronic supplementary material:**

The online version of this article (10.1186/s40425-018-0391-1) contains supplementary material, which is available to authorized users.

## Background

The incidence of malignant melanoma has been on the rise over the past few decades. An estimated 351,880 new cases of melanoma have been diagnosed worldwide in 2015 with a mortality rate of 17% [1]. Less than a decade ago, the treatment options were very limited for patients with advanced stage disease and the 5-year overall survival (OS) was only 9–28% [2, 3]. With the development of immunotherapies as well as targeted therapies, the OS has significantly improved. Currently, the known 3-year OS for patients with stage IV melanoma reaches up to 58% [4]. In spite of these recent clinical successes, still a large group of patients fail to respond to therapy or progress after initial response, which brings the need for additional treatment modalities.

One such additional treatment option is adoptive cell therapy (ACT) with tumor-infiltrating lymphocytes (TIL). ACT with TIL has been of growing interest as anti-cancer treatment in the past decade. This therapy consists of the outgrowth of tumor resident T cells from tumor material, their expansion ex vivo and transfer back into the same patient after a lymphodepleting preparative regimen [5]. In many studies, the infused T cells are supported by high-dose interleukin-2 (HD IL-2) to facilitate engraftment of the cells.

After the first demonstration of promising clinical effects of TIL in melanoma patients in the 90’s and the beginning of the new millennium by the Surgery Branch of the National Institutes of Health (SB, NIH, Bethesda, Maryland, US) [6–8], multiple clinical trials at different sites over the world confirmed these results. In these trials, objective responses varying between 40 and 70% have consistently been observed [8, 9]. As the applicability and scope of ACT with TIL is broadening, the optimization of TIL production, including selection of T cell subsets, and adjustment of the clinical protocol, including the lymphodepleting preparative regimens and the role of IL-2 are of utmost importance. Of upcoming interest is also the potency of TIL transfer in adjuvant setting [10], as combination therapy [11], as well as its efficacy in other solid tumors [12–14].

In this review, we will provide an overview of the current state of ACT with TIL in melanoma, focusing on clinical responses, production and treatment protocols, associated toxicity, as well as the future potential of TIL therapy as anti-tumor treatment.

## Current state of TIL treatment in malignant melanoma

The first objective clinical responses with TIL treatment were seen in a series of phase I/II trials, all executed by Rosenberg and colleagues more than 20 years ago at the NIH, in which infusion of TIL was combined with lymphodepleting conditioning regimes and HD IL-2 [6–8]. Consistent objective response rates (ORR) up to 72% were reached with TIL therapy in several consecutive clinical trials, in which 10–20% of treated patients reached a complete remission (CR) and 40% of patients achieved durable clinical responses. These durable responses were predominantly seen in patients who achieved CR at an early time point and the chance for response did not seem to be influenced by progression upon prior systemic treatment [8, 9, 15–19]. Objective responses seemed to be associated with higher number of infused cells [18].

Originally, the conditioning non-myeloablative (NMA) regimen consisted of cyclophosphamide (60 mg/kg) for 2 days, followed by fludarabine (25 mg/m^2^) for 5 days. The infusion of TIL products followed > 24 h after the final dose of fludarabine. Patients subsequently received HD IL-2 (720,000 IU/kg intravenously (i.v.) every 8 h up to 15 doses or until intolerance [6, 8, 16]. Other trials have been conducted with adjusted production protocols, different conditioning regimens, and IL-2 schedules, which will be discussed below.

The encouraging results of TIL therapy in melanoma have stimulated centers worldwide to conduct studies in order to reproduce and optimize this treatment. Focus for optimization was directed at cell fraction, preparative regimen and IL-2 dose. Additional file 1: Table S1 shows an overview of these studies. The conducted studies with TIL in patients with metastatic melanoma have predominantly been as first-line treatment or in patients with progression upon prior systemic immunotherapy. These treatments mostly consisted of chemotherapy with dacarbazine, interferon-α, IL-2, ipilimumab, an anti-CTLA-4 antibody, or combinations [8, 15, 16, 18, 19]. Treatment with PD-1 blockade, or an anti-PD-1-based combination, is now mostly first-line therapy in patients with advanced melanoma, showing an unprecedented 3-year overall survival around 50% [4]. The role of TIL as possible first-line therapy in combination with anti-PD-1 is currently subject of clinical trials, and one has to await the first results to estimate the additive effect of TIL and anti-PD-1. However, TIL therapy may be a potential option in patients with disease progression upon PD-1 blockade, as current treatment options for these patients is still very limited. Whether TIL should be given in combination with anti-PD-1 or as a single treatment option is still an unknown. To provide evidence that TIL therapy is more effective than the current standard of care with anti-CTLA-4 (ipilimumab) for patients with advanced melanoma upon progression on up to one prior treatment, a multicenter randomized phase III trial is actively recruiting patients at the Netherlands Cancer Institute (NKI, Amsterdam, The Netherlands) and the Center for Cancer Immune Therapy (CCIT, Herlev, Denmark). The patients enrolled in this trial are randomized in a 1:1 ratio between ipilimumab and TIL treatment (NCT02278887). Currently, the vast majority of patients that are enrolled in this trial have progressed on anti-PD-1 treatment. In addition to this phase III trial, another 22 clinical trials worldwide are being performed with TIL therapy in melanoma to evaluate the optimal treatment form, with varying TIL production and treatment protocols, and as combination therapy. For a complete overview of these clinical trials, see Table 1.

**Table 1:** Current Trials with Tumor-infiltrating Lymphocytes in Melanoma Registered by ClinicalTrials.gov per March 2018

Trial	Institute	Phase	Estimated enrollment	Intervention	TIL product	Lymphodepletion regimen	IL-2 regimen	Disease Stage	Primairy Outcome Measures	Identification number
a. Recruiting Trials										
Combined Therapy of Nivolumab and Adoptive T Cell Therapy in Metastatic Melanoma Patients: Pilot Study Phase I/II	Nantes University Hospital, Nantes, France Initiation 2018	I/II	11	TIL + IL-2 + Nivo (3 mg/kg every 2 w until w52)	Cohort 1: 5 × 10^8^ TIL (3 patients) Cohort 2: 1-20 × 10^9^ TIL at 14 w and 18 w	Not described	600,000 IU/kg/d for 5d	Stage IIIb, IIIc or IV melanoma	AE	NCT03374839
Phase I Study to Assess Feasibility and Safety of Adoptive Transfer of Autologous Tumor-infiltrating Lymphocytes in Combination With Interleukin-2 Followed by Nivolumab Rescue for Advanced Metastatic Melanoma	CHUV, Lausanne, Switzerland Initiation 2018	I	10	Lymphodepletion + TIL + IL-2 +/− Nivo (3 mg/kg, every 2w max 24 months) rescue	Unspecified	Cy i.v. for 2d and Flu i.v. 5d (not otherwise specified)	HD IL-2 t.i.d. max 8 doses	Stage IV melanoma	Feasibility AE	NCT03475134
A Phase 2, Single-Center, Open Label Study of Autologous, Adoptive Cell Therapy Following a Reduced Intensity, Non-myeloablative, Lymphodepleting Induction Regimen in Metastatic Melanoma Patients	Sheba Medical Center, Israel Initiation 2017	II	30	Lymphodepletion + TIL + IL-2	Unspecified	Flu (25 mg/m^2^ for 3 d) + TBI (2 Gy as single treatment)	720,000 IU/kg t.i.d.. until tolerable toxicity, max 10 doses	Measurable metastatic melanoma	ORR AE	NCT03166397
Phase Ib Trial of Pembrolizumab Administered in Combination With or Following Adoptive Cell Therapy- A Multiple Cohort Study; The ACTIVATE (Adoptive Cell Therapy InVigorated to Augment Tumor Eradication) Trial	Princes Margaret Cancer Centre, Canada Initiation 2017	Ib	24	Cohort 1: Lymphodepletion + TIL + IL-2 + pembro (200 mg q 3 w) Cohort 2: Lymphodepletion + TIL + IL-2 + pembro (200 mg q 3 w)	1 × 10^10^–1.6 × 10^11^ TILs	Cohort 1: Cy i.v. 60 mg/kg/d for 2d + Flu 25 mg/m^2^ for 5 d Cohort 2: Cy i.v. 30 mg/kg per day for 2 days	Cohort 1 + 2: 125,000 IU/kg s.c./d	Unresectable stage III/ IV melanoma or Platinum resistant ovarian cancer	AE	NCT03158935
A Pilot Clinical Trial Combining PD-1 Blockade, CD137 Agonism and Adoptive Cell Therapy for Metastatic Melanoma	Lee Moffitt Cancer Center, Florida, US Initiation 2016	Pilot	12	Cohort 1 (1st 6 patients): Lymphodepletion + TIL + IL-2 Cohort 2 (2nd 6 patients): Pre-treatment nivo + lymphodepletion + TIL + IL-2	Unspecified, outgrowth in 4–8 w with CD137 activating antibody	Cy 2 d beginning 3–6 w after tumor collection for TIL growth + Flu for 5 d	Unspecified	Unresectable cutaneous or mucosal stage III/IV melanoma	AE Feasibility	NCT02652455
A Prospective Randomized and Phase 2 Trial for Metastatic Melanoma Using Adoptive Cell Therapy With Tumor Infiltrating Lymphocytes Plus IL-2 Either Alone or Following the Administration of Pembrolizumab	NIH Clinical Center, Bethesda, Maryland, US Initiation 2015	II	170	Cohort 1 Arm 1 Anti-PD1/PD-L1 refractory patients: Lymphodepletion + TIL + IL-2 Cohort 1 Arm 2 Anti-PD1/PD-L1 refractory patients: Lymphodepletion + Pembro 2 mg/kg i.v. on d − 2, (and d 21 (+/− 2 d), 42 (+/− 2 d), and 63 (+/− 2 d) following cell infusion) + TIL + IL-2 Cohort 2 Arm 3 Anti-PD1/PD-L1 naive patients: Lymphodepletion + Pembro 2 mg/kg i.v. on d-2 (and days 21 (+/− 2 d), 42 (+/− 2 d), and 63 (+/− 2 d)) following cell infusion + TIL + IL-2 Cohort 1 Arm 1 (Retreatment) Anti-PD1/PD-L1 refractory patients with no response to study treatment or PD after PR/CR: Pembro 2 mg/kg i.v. on d − 2, and d 21 (+/− 2 d), 42 (+/− 2 d), and 63 (+/− 2 d)	Young TIL, not otherwise specified	Cohort 1 + 2: Cy 60 mg/kg i.v. for 2d + Flu 25 mg/m^2^for 5d	Cohort 1 + 2: 720,000 IU/kg i.v. t.i.d., max 12 doses	Measurable metastatic melanoma	ORR	NCT02621021
A Phase 2, Multicenter Study to Assess the Efficacy and Safety of Autologous Tumor Infiltrating Lymphocytes (LN-144) for Treatment of Patients With Metastatic Melanoma	Iovance Investigative Site, Los Angeles, California, US Initiation 2015	II	60	Cohort 1: Lymphodepletion + TIL + IL-2 Cohort 2: Lymphodepletion + TIL + IL-2 Cohort 3: re-treatment lymphodepletion + TIL + IL-2	Cohort 1: LN-144 autologous TIL non-cryopreserved product Cohort 2: LN-144 autologous TIL cryopreserved Cohort 3: LN-144 autologous TIL re-treatment for 2nd LN-144 infusion	Lymphodepleting chemotherapy, not otherwise specified	Unspecified	Unresectable metastatic melanoma	ORR	NCT02360579
A Pilot Study of Lymphodepletion Plus Adoptive Cell Transfer With T-Cells Transduced With CXCR2 and NGFR Followed by High Dose Interleukin-2 in Patients With Metastatic Melanoma	MD Anderson Cancer Center, Houston, Texas, US Initiation 2015	Pilot	15	Lymphodepletion + transduced TIL + IL-2	Up to 1.5 × 10^11^ TIL (CXCR2 and NGFR transduced TIL)	Cyc 60 mg/kg for 2d + Flu 25 mg/m^2^for 5d	720,000 IU/kgi.v. every 8–16 h, max 15 doses	Metastatic melanoma or stage III in-transit, subcutaneous, or regional nodal disease	AE	NCT01740557
T-cell Therapy in Combination With Vemurafenib for Patients With BRAF Mutated Metastatic Melanoma	CCIT, Copenhagen, Herlev, Denmark Initiation 2014	I/II	12	Vem 960 b.i.d. 7d before tumor harvest until lymphodepletion (d − 8) + TIL + IL-2	4-6 weeks culture time Infusion 1 × 10^9^-2 × 10^11^ TIls	Cy 60 mg/kg for 2d + Flu 25 mg/m^2^ for 5 d	Decrescendo regimen (18 MIU/m2 for 6 h, 18 MIU/m2 for 12 h, 18 MIU/m2 for 24 h followed by 4,5 MIU/m2 for another 3 × 24 h)	Unresectable stage III/IV melanoma	AE	NCT02354690
Randomized Phase III Study Comparing a Non-myeloablative Lymphocyte Depleting Regimen of Chemotherapy Followed by Infusion of Tumor Infiltrating Lymphocytes and Interleukin-2 to Standard Ipilimumab Treatment in Metastatic Melanoma	CCIT, Copenhagen, Herlev, Denmark NKI, Amsterdam, Netherlands Initiation 2014	III	168	Cohort 1: Ipi 4 cycles (i.v. 3 mg/kg q 3 weeks) Cohort 2: Lymphodepletion + TIL + IL-2	Unspecified	Cy 60 mg/kg iv for 2d + Flu 25 mg/m^2^ for 5d	600,000 IU/kg t.i.d., max 15 doses	Unresectable stage III/IV melanoma	PFS	NCT02278887
A Pilot Study of Lymphodepletion Plus Adoptive Cell Transfer With TGF-beta Resistant (DNRII) and NGFR Transduced T-Cells Followed by High Dose Interleukin-2 in Participants With Metastatic Melanoma	MD Anderson Cancer Center, Houston, Texas, US Initiation 2014	Pilot	15	Lymphodepletion + transduced TIL + IL-2	Transduced DNRII TIL, equal number of transduced NGFR TIL, up to a total of 1.5 × 10^11^ TIL	Cy 60 mg/kg i.v. for 2d + Flu 25 mg/m^2^ i.v. for 5d	720,000 IU/kg i.v. every 8–16 h max 15 doses on d 1–5 + 22–26	Metastatic melanoma or stage III in-transit, subcutaneous, or regional nodal disease (turnstile I)	Feasibility	NCT01955460
A Phase II Study for Metastatic Melanoma Using High Dose Chemotherapy Preparative Regimen Followed by Cell Transfer Therapy Using Tumor Infiltrating Lymphocytes Plus IL-2 With the Administration of Pembrolizumab in the Retreatment Arm	NIH, Bethesda, Maryland, US Initiation 2013	II	64	Cohort 1: Lymphodepletion + TIL + IL-2 Cohort 2 Retreatment Arm: 4 doses pembro Non-responders of patients with PR/CR and progress with prior pembro/nivo treatment may receive a second treatment. D 0 (2–4 d after last dose of flu), Pembro 2 mg/kg i.v. +/− 4 h prior to cell infusion. D 21 (+/− 2 d) following cell infusion, Pembro 2 mg/kg i.v. D 42 (+/− 2 d) following cell infusion, Pembro 2 mg/kg IV. D 63 (+/− 2 d) following cell infusion, Pembro 2 mg/kg i.v.	Young TIL	Cy 60 mg/kg/day for 2 d + Flu 25 mg/m^2^ i.v. for 5 d	720,000 IU/kg i.v. t.i.d., max 12 doses	Measurable metastatic melanoma	ORR	NCT01993719
A Phase I Study to Evaluate Safety, Feasibility and Immunologic Response of Adoptive T Cell Transfer With or Without Dendritic Cell Vaccination in Patients With Metastatic Melanoma	Karolinska University Hospital Stockholm, Sweden Initiation 2013	I	10	Cohort 1: Lymphodepletion + TIL + IL-2 Cohort 2: Lymphodepletion + TIL + IL-2 + i.d. DC vaccinations with up to 1.5 × 10^7^ DC pulsed with autologous tumor lysate and NY-ESO-1 peptide after completion of IL-2	Up to 5 × 10^10^ TILs i.v. infusion	Cy 60 mg/kg i.v. (d − 7&-6) + Flu 25 mg/m^2^ i.v. (d − 5 to − 1)	100,000 IU/kg t.i.d., maximum 14 doses	Inoperable stage III or stage IV melanoma	Safety	NCT01946373
Phase II Study Evaluating The Infusion Of Autologous Tumor-Infiltrating Lymphocytes (TILs) And Low-Dose Interleukin-2 (IL-2) Therapy Following A Preparative Regimen Of Non-Myeloablative Lymphodepletion Using Cyclophosphamide And Fludarabine In Patients With Metastatic Melanoma	Princess Margaret Cancer Centre Toronto, Ontario, Canada Initiation 2013	II	12	Lymphodepletion + TIL + IL-2	1 × 10^10^–1.6 × 10^11^ TILs	Cy 60 mg/kg i.v. for 2 d + Flu 25 mg/m^2^ i.v. for 5 d	125,000 IU/kg/d for 2 w (2 d rest between each w)	Measurable, unresectable stage III/IV melanoma	ORR	NCT01883323
Lymphodepletion Plus Adoptive Cell Transfer With or Without Dendritic Cell Immunization in Patients With Metastatic Melanoma	MD Anderson Cancer Center Houston, Texas, US Initiation 2006	II	189	Cohort 1: Lymphodepletion + TIL + IL-2 Cohort 2: Lymphodepletion + TIL infusion + IL-2 + 1 × 10^7^–2.5 × 10^8^ MART-1 peptide-pulsed DC i.v. Cohort 3 Prior treatment with BRAF-inhibitor: Lymphodepletion followed by TIL + IL-2 + 1 × 10^7^–2.5 × 10^8^MART-1 peptide-pulsed DC i.v. Cohort 4 Leptomeningeal Disease: TIL d 1 + d 15	Cohort 1–3: Up to 1.5 × 10^11^ TIL Cohort 4: 5.0 × 10^9^ TIL on d 1 + 10 × 10^9^TIL on d 15	Cy 60 mg/kg for 2 d + Flu 25 mg/m^2^ for 5d	Cohort 1–3: 720,000 IU/kg every 8–16 h, max doses on d 1–5 + 22–26 (+/− 7 d), as tolerated Cohort 4: 1.2 MIU of IL- 2 on d 2, 4, 9, 11, 16 and 18 as tolerated. Subsequently 2×/w IL-2 to weekly IL-2. After 4–6 w IL-2 maintenance	Metastatic melanoma, uveal melanoma or stage III in-transit or regional nodal disease	ORR	NCT00338377
b. Trials not yet recruiting										
A Phase 2 Study to Evaluate the Efficacy and Safety of Adoptive Transfer of Autologous Tumor Infiltrating Lymphocytes in Patients With Metastatic Uveal Melanoma	UPMC Hillman Cancer Center, Pittsburgh, Pennsylvania, US Initiation 2018	II	59	Lymphodepletion + TIL + HD IL-2	1 × 10^9^ - 2 × 10^11^ TIL per current standard protocol	Cy and Flu (not otherwise specified)	600,000 IU/kg t.i.d. max 6 doses	Measurable metastatic uveal melanoma	ORR	NCT03467516
A Randomised Phase II Study in Metastatic Melanoma to Evaluate the Efficacy of Adoptive Cellular Therapy With Tumor Infiltrating Lymphocytes (TIL) and Assessment of High Versus Low Dose Interleukin-2	The Christie NHS Foundation Trust, Manchester, UK Initiation 2013	II	90	Arm A: lymphodepletion + TIL + HD IL-2 Arm B: lymphodepletion + TIL + LD IL-2	Unscpecified	Cy 60 mg/kg 2 d + Flu 25 mg/m^2^5 d	Arm A: HD IL-2, max 12 doses Arm B: LD IL-2, max 12 doses	Metastatic melanoma	ORR	NCT01995344
c. Non-recruiting Trials										
T Cell Therapy in Combination With Peginterferon for Patients With Metastatic Melanoma	CCIT, Copenhagen, Herlev, Denmark Initiation 2014	I/II	12	Lymphodepletion + TIL + IL-2 + s.c. injections of peginterferon- α 3× (d − 2, d 7 and d 14)	4-6 weeks culture time Maximum number of TILs	Cy 60 mg/kg i.v for 2d + Flu 25 mg/m^2^ i.v for 5d	Continuous infusion decrescendo regimen (18 MIU/m^2^ IL-2 over 6 h, 18 MIU/m^2^ IL-2 over 12 h, 18 MIU/m^2^ IL-2 over 24 h followed by 4.5 MIU/m^2^ IL-2 over 24 h for 3d	Unresectable stage III/IV melanoma	AE	NCT02379195
Cellular Adoptive Immunotherapy Using Autologous Tumor-infiltrating Lymphocytes Following Lymphodepletion With Cyclophosphamide and Fludarabine for Patients With Metastatic Melanoma	University of Washington Cancer Consortium, Seattle, Washington, US Initiation 2013	II	13	Lymphodepletion + TIL + IL-2	Unspecified	Cy for 2d + Flu for 5d (not otherwise specified)	Unspecified	Stage III/IV melanoma	ORR	NCT01807182
Co-stimulation With Ipilimumab to Enhance Lymphodepletion Plus Adoptive Cell Transfer and High Dose IL-2 in Patients With Metastatic Melanoma	Moffitt Cancer Center and Research Institute, Tampa, Florida, US Initiation 2012	Pilot	13	Pre-treatment with ipi (cycle 1) prior to surgery to retrieve TILs. Cycle 2 of ipi 1 w after surgery (3 w after 1st cycle) followed by Lymphodepletion + TIL + IL-2	6 weeks outgrowth	Cy for 2d + Flu for 5d (not otherwise specified)	HD IL-2, otherwise unspecified. T.i.d., max 15 doses	Unresectable stage III/IV melanoma	Safety Feasibility	NCT01701674
Phase II Clinical Trial of Vemurafenib With Lymphodepletion Plus Adoptive Cell Transfer and High Dose IL-2 in Patients With Metastatic Melanoma	Moffitt Cancer Center and Research Institute, Tampa, Florida, US Initiation 2012	II	17	Vem (3w prior to TIL + post TIL for 2 yr) followed by Lymphodepletion + TIL infusion + IL-2	Unspecified	Cy for 2d + Flu for 5d (not otherwise specified)	HD IL-2 (not otherwise specified)	Unresectable metastatic stage IV melanoma or stage III intransit or regional nodal disease	ORR Dropout rate	NCT01659151
Prospective Randomized Study of Cell Transfer Therapy for Metastatic Melanoma Using Tumor Infiltratring Lymphocytes Plus IL-2 Following Non-Myeloablative Lymphocyte Depleting Chemo Regimen Alone or in Conjunction With 12Gy Total Body Irradiation (TBI)	NIH, Bethesda, Maryland, US Initiation 2011	II	102	Cohort 1: Lymphodepletion + TIL + IL-2 Cohort 2: Lymphodepletion followed by TBI + TIL + IL-2	Cohort 1 + 2: 1 × 10^9^-2 × 10^11^young TILs	Cohort 1 + 2: Cy 60 mg/kg for 2 d + Flu 25 mg/m^2^ for 5 d Cohort 2: 2Gy of TBI 2×/day for 3d (total dose 12Gy) 3d prior to TIL infusion	Cohort 1 + 2: 720,000 IU/kg i.v. t.i.d., max 15 doses	Measurable metastatic melanoma	ORR	NCT01319565
Lymphodepletion Plus Adoptive Cell Transfer With High Dose IL-2 in Patients With Metastatic Melanoma	Moffitt Cancer Center, Tampa, Florida, US Initiation 2009	I/II	19	Lymphodepletion + TIL + IL-2	Unspecified	Cyc 60 mg/kg for 2d + Flu 25 mg/m^2^ for 5d	720,000 IU/kg i.v. t.i.d max 15 doses	Unresectable stage III/IV melanoma	Feasibility	NCT01005745

*Abbreviations*: *AE* adverse event, *b.i.d*. bis in die, *CCIT* Center for Cancer Immune Therapy, *CD* Cluster of differentiation, *CHUV* Centre hospitalier universitaire Vaudois, *CR* complete response, *CXCR* C-X-C chemokine receptor, *Cy* cyclophosphamide, *d* day, *DC* dendritic cell, *Flu* fludarabine, *Gy* Gray, *HD* high-dose, *hr* hour, *i.d* intradermal, *i.v*. intravenous, *IL-2* interleukin-2, *Ipi* ipilimumab, *IU* international unit, *kg* kilogram, *LD* low dose, *LN-144* TIL production technology developed by Iovance Biotherapeutics, *MART-1* Melanoma antigen recognized by T cells 1, *max* maximum, *mg* milligram, *NA* not available, *NGFR* nerve growth factor receptor, *NHS* National Health Service, *NIH* National Institutes of Health, *Nivo* nivolumab, *NKI* National Cancer Institute, *ORR* objective response rate, *PD* progressive disease, *PD-1* Programmed cell death protein-1, *PDL-1* Programmed death ligand-1, *Pembro* pembrolizumab, *PFS* progression free survival, *PR* partial response, *q* every, *RR* response rate, *s.c.* subcutaneous, *t.i.d*. ter in die, *TBI* total body irradiation, *TIL* tumor-infiltrating lymphocytes, *UK* United Kingdom, *UPMC* Universite Pierre and Marie Curie, *US* United States, *Vem* vemurafenib, *w* week, *x* times, *yr* year

### Evidence for lymphodepleting preparative regimens

The necessity of temporary lymphodepleting preconditioning before TIL infusion remains an important aspect in ACT with TIL. The first evidence for the need of lymphodepletion with either chemotherapy or total body irradiation (TBI) was demonstrated in murine models, where improved response rates were seen with TIL after lymphodepletion [20, 21]. Lymphodepletion with either TBI or NMA chemotherapy is thought to improve the effector function of TIL in several ways. Firstly, data from several studies suggest that the endogenous subpopulation of CD4^+^CD25^+^ regulatory T cells (Tregs) capable of suppressing immune responses may be depleted [22]. Secondly, lymphodepletion of the host reduces the pool of endogenous lymphocytes competing with the transferred T cells for homeostatic cytokines, especially IL-7 and IL-15 [23]. These cytokines are produced by non-lymphoid sources in response to lymphopenia, where IL-7 is required for the proliferation and survival of T cells and IL-15 maintains and improves the proliferation of the T cells [24, 25]. Lastly, lymphodepletion is believed to generate “physical space” for the infusion product.

In 2002, the Surgery Branch of the NIH demonstrated the clinical importance of lymphodepletion before TIL infusion. In this study, 13 patients with metastatic melanoma were treated with cyclophosphamide 60 mg/kg/day for 2 days and fludarabine 25 mg/m^2^/day for 5 days prior to TIL infusion and achieved an ORR of 46% [7], which compared favorable to response rates of 31% without prior lymphodepletion [6]. In 2008, this same group examined the effect of intensifying the lymphodepleting regimen by adding TBI to the above mentioned NMA chemotherapy and improved clinical outcomes with this strategy. Patients were treated with cyclophosphamide and fludarabine with addition of either 2 Gy or 12 Gy TBI, with 25 patients in each cohort. Compared to the cohort treated solely with chemotherapy showing an ORR of 49%, addition of TBI with 2 Gy or 12 Gy improved these ORR to 52 and 72% respectively [16].

In a follow-up randomized trial, the additional benefit described above of TBI in addition to NMA chemotherapy for the ORR could not be confirmed. A total of 101 patients with metastatic melanoma were either treated with NMA chemotherapy as described above per standard protocol, or in combination with 1200 cGy TBI (TBI 2 Gy twice a day for 3 days) prior to TIL infusion. Clinical outcome was similar in both treatment arms, with durable CR seen in 24% of patients in both cohorts and no significant difference in ORR of 45 and 62% in patients pretreated with NMA chemotherapy alone or with the addition of TBI respectively (*p* = 0.11). Of note, addition of TBI did result in additional toxicity, namely thrombotic microangiopathy in 27% of patients [26].

Temporary lymphodepletion with chemotherapy, TBI or a combination thereof appear to have an additive effect on the efficacy of TIL therapy as described above. Nevertheless, the question remains what the most optimal regimen is, in terms of both duration and depth of lymphodepletion and in terms of which drug(s) to use. Answers to these questions are not only relevant to further improve response rate to TIL therapy, but also to minimize toxicities, now predominantly consisting of transient pancytopenia and febrile neutropenia occurring in 37–96% of patients [18, 19].

To address these questions, the Sheba Medical Center, Israel, is currently conducting a phase II clinical trial exploring the efficacy of reduced intensity lymphodepletion using fludarabine 25 mg/m^2^ for 3 days (instead of five per standard protocol and no addition of cyclophosphamide) followed by TBI (2 Gy single treatment for 1 day prior to TIL infusion (NCT03166397). This clinical trial is still recruiting and is expected to give more insight into the optimal lymphodepleting regimen prior to the infusion of TIL in melanoma patients.

### The role of interleukin-2 in the current treatment protocol

Single agent IL-2 has received approval by the US Food and Drug Administration for the treatment of metastatic renal cell cancer and metastatic melanoma in 1992 [27] and 1998 [28] respectively. When used in combination with TIL, IL-2 is thought to enhance the anti-tumor response by continuous support of growth and activity of the infused TIL products. Studies suggest that IL-2 may enhance the inherent antitumor activity of CD8^+^ T cells and the cytolytic function of natural killer cells [29]. However, IL-2 is also associated with a variety of toxicities, some associated with capillary leak syndrome presented by edema, hypotension and reduced urine output within hours of infusion, but also fevers, rigors, myalgia and nausea. Most of these toxicities can be managed well by supportive measures [28]. However, so far no clear correlation between the number of IL-2 infusions and clinical response could be demonstrated. It is therefore worthwhile to reconsider the role of HD IL-2 administration in combination with TIL infusion.

A phase I trial at the NIH evaluated the anti-tumor effect of TIL therapy with varying IL-2 doses ranging from 0 to 720,000 IU/kg in 15 patients with metastatic melanoma. Patients receiving either low-dose (LD) IL-2 (72,000 IU/kg i.v. every 8 h up to 15 doses) (*n* = 3) or HD IL-2 (720,000 IU/kg i.v. every 8 h up to 12 doses) (*n* = 6) following NMA chemotherapy and infusion of TIL showed reduction in tumor volume. This effect was not seen in patients that did not receive any IL-2 (*n* = 6) [30]. Of importance, however, is that these findings are based on a small study and confirmation of these data would require a larger prospective trial. The CCIT, Herlev, Denmark, demonstrated clinical responses in patients with metastatic melanoma treated with lymphodepleting chemotherapy and TIL infusion followed by subcutaneous (s.c.) LD IL-2 injections (2 MIU for 14 days). Durable objective responses were seen in 2/6 (33%) patients and 2/6 (33%) patients showed disease stabilization [31]. In another phase I/II study by the same group, administration of intravenous IL-2 in a decrescendo regime also showed clinically significant responses with an ORR of 42%. In this trial, 25 patients with metastatic melanoma were treated with standard lymphodepleting chemotherapy and TIL infusion followed by 5 days of continuous infusion of IL-2 in a decrescendo manner, with 18 MIU/m^2^ over 6, 12 and 24 h followed by 4.5 MIU/m^2^ over 24 h for 3 days [18]. These data from the NIH and CCIT suggest that it might be possible to lower the dose of IL-2, without negatively effecting clinical outcome.

Currently, several clinical trials are being conducted to evaluate the clinical efficacy of these different IL-2 regimens in ACT with TIL, as presented in Table 1.

### Toxicity

The most common toxicities during TIL therapy are due to the effects of the lymphodepleting preparative regimens and the subsequent IL-2 after TIL infusion [32]. TIL-related toxicity is less common, but patients may develop, mostly transient, dyspnea, chills and fever shortly after infusion of TIL. Other signs of toxicity develop later after infusion and may consist of melanoma associated autoimmune diseases such as vitiligo or uveitis, of which the latter promptly responds to topical corticosteroid treatment. This demonstration of autoimmune-like toxicity does not seem to be significantly correlated with response to TIL therapy [19]. In general, autoimmune-like toxicity such as uveitis, hearing loss and vitiligo after TIL therapy is much less common compared to development of these side-effects following ACT with MART-1 or gp100 specific T cell receptor (TCR) gene therapy [33]. One plausible reason for this difference could be that TIL products consist of a more polyclonal T cell population targeting more and other antigens than the homogenous T cell population in the TCR gene therapy product.

Autoimmune toxicity due to TIL therapy is not always transient, as described by Yeh et al. In this case report, a patient undergoing TIL therapy developed severe autoimmune sequelae including diffuse erythematous full-body rash, persistent panuveitis and hearing loss. The patient was treated following preparative lymphodepletion with cyclophosphamide, fludarabine and TBI 12 Gy prior to infusion of 1.4 × 10^11^ autologous TIL and 4 doses of HD IL-2. Biopsy of the rash showed dermal CD8^+^ T cell infiltrates. Flow cytometry of ex vivo expanded T cells from biopsies of the eyes demonstrated much higher percentages MART-1 MHC multimer-positive CD8^+^ cells compared to the peripheral blood after TIL therapy. The patient showed a durable CR of the metastatic melanoma 2 years after TIL therapy [34]. Although this case report suggests a positive correlation between the occurrence of autoimmune toxicity and response to ACT with TIL, such a correlation has not yet been demonstrated in larger patient cohorts.

## Characterization of TIL products

The clinical efficacy of TIL therapy is greatly dependent on the specific quality of T cells to recognize and eradicate the tumor cells. In previous trials using TIL therapy in patients with metastatic melanoma, a significant correlation was seen between clinical benefit and culture time, percentage of CD8^+^ and CD8^+^/CD27^+^ cells and the absolute number of infused tumor reactive T cells in the in the infusion product [8, 17, 18, 35]. When autologous tumor material from patients is available, one can test the tumor reactivity of the generated TIL product in vitro by co-culture of the TIL with the autologous tumor cell lines or tumor digest, with as read-out the production of effector cytokines, such as IFN-γ and TNF-α, or degranulation markers (such as CD107) by the T cells. In our experience, up to 30% of the T cells in the infusion products are able to recognize autologous tumor material (unpublished data).

A substantial amount of cross-institutional effort has been made over the past decades to dissect what tumor-reactive T cells in TIL products recognize on human melanoma. To define the characteristics of potential T cell targets on melanoma, melanoma antigens can be separated in two major categories: Tumor-associated and tumor-specific antigens. The tumor-associated antigens include self-antigens with an aberrant expression in cancer, such as overexpressed (OE) antigens and cancer/testis (C/T) antigens, as well as tissue-specific antigens such as the melanoma differentiation (MD) antigens. These antigens are shared between subgroups of patients. The tumor-specific antigens include viral antigens in cancers associated with viral infections such as human papillomavirus (HPV) positive tumors where the oncogenes are viral proteins and the mutated antigens (neo-antigens), which arise as a consequence of DNA damage.

For decades, we have known that tumor-specific T cells in melanoma frequently recognize MD antigens. In particular MART-1 and gp100 are found to be recognized by the vast majority of TIL products. In previous work we have detected MART-1 specific T cell responses in 27 of 34 TIL infusion products, and gp100 specific T cell responses in 14 of these products [36]. Neo-antigens are conceptually very interesting T cell targets as they have the potential to be as foreign as for example, viral antigens because central tolerance is not expected. The first evidence for T cells recognizing neo-antigens was provided in a landmark study from 1995 by Wölfel et al., in which a T cell clone isolated from a melanoma patient was shown to recognize a mutated version of CDK4 [37]. At that point in time, technical limitations prevented a more systematic assessment of such T cell responses. Now, some 20 years later, technological advances in the ability to perform high throughput exome sequencing, together with the development of screening platforms for antigen-specific T cells, have made it realistic to investigate the frequency with which T cells recognize mutated antigens. Seminal work from the groups of Schreiber [38] and Sahin [39] provided proof of concept that these newly-developed technologies could be utilized to dissect T cell responses towards neo-antigens in their mouse models. Subsequently, work from multiple groups including ours, has cemented the paradigm that TIL frequently respond to neo-antigens in human melanoma. Furthermore, patients with tumors baring high mutational load are more likely to clinically benefit from immunotherapy than those with tumors with a low mutational burden. This has been demonstrated for lung cancer patients treated with anti-PD-1 therapy [40], melanoma patients treated with anti-CTLA-4 [41], and patients with mismatch repair deficient tumors across several cancer types with anti-PD-1 treatment [42, 43]. Based on these observations, it seems likely that T cells specific for neo-antigens play an important role in the responsiveness to immunotherapy. Direct evidence that neo-antigen specific T cells can be clinically relevant was provided by the group of Rosenberg and colleagues. They showed that patients can experience tumor regression upon adoptive cell treatment with T cell products enriched for neo-antigen specific T cells [44]. In addition, we have detected multi neo-antigen directed T cells in our in-house produced TIL products (van den Berg et al., manuscript in preparation). Together, these findings make it attractive to enrich for neo-antigen specific T cell reactivity in TIL therapy to increase the response rate.

## TIL production

In general, TIL production can be divided into two steps; initial outgrowth and rapid expansion (REP). Initial out-growth starts with the excision of a melanoma metastasis of at least 2–3 cm, followed by transport to a Good Manufacturing Practice (GMP) production facility. This metastasis (or multiple smaller metastases) are cut into small pieces (of a few millimeters) or enzymatically digested into a single cell suspension. Fragments or digests are then cultured in the presence of IL-2 to allow the outgrowth of TILs. Initial outgrowth takes around 14 days for an average TIL patient (range at NKI 7 to 21 days), resulting in at least 50 × 10^6^ TILs. During the outgrowth of a digest, tumor cells disappear from the cultures. The use of tumor fragments [17, 18] or digest [45, 46] seem not to influence the success rates of TIL outgrowth and/or clinical response.

During REP, which takes 14 days in the standard protocol, TILs are stimulated and further expanded to large numbers (between 1 × 10^10^ and 2 × 10^11^ cells). At the start of a REP, soluble anti-CD3 antibody, irradiated feeder cells (from autologous or allogenic source) in a 100–200 fold excess to the TILs, and IL-2 is added to the T cells. These irradiated feeders release growth factors into the culture which will accommodate massive TIL expansion, usually more than 1000-fold. During the last phase of the REP, a bioreactor (such as WAVE or Xuri, or gas permeable G Rex bottles) is required to allow culture of high cell densities [47]. The current success rate of TIL outgrowth is very high, although not 100%. The group at the NIH achieved to grow viable TIL in 75–85% from 93 melanoma patients [8] and the CCIT in Denmark recently reported a success rate of 97% (32 out of 33 TIL cultures from melanoma patients) [18].

Whether IL-2 is the most optimal cytokine for the outgrowth phase of TIL is debatable. It is known that IL-2 leads to the terminal effector state of T cells. This, in combination with the long production time of TIL, makes the final infusion product consist mainly of exhausted T cells. It would make sense to switch to homeostatic cytokines such as IL-7, IL-15 and IL-21 to generate a less differentiated T cell product. This could possibly result in longer engraftment and better tumor control in the recipient, as has already been observed in animal models [48]. However, since clinically active TIL production protocols are currently based on the use of IL-2, it is difficult to switch. Ideally, a clinical trial comparing different cytokine combinations for TIL outgrowth should be conducted to provide clarity about which cytokine strategy is superior.

### “Young” TILs

In most early studies, several TIL cultures were established per patient and only the tumor reactive cultures were pre-selected for further outgrowth. Tumor reactivity was detected based on IFN-γ production upon in vitro co-culturing with autologous tumor material or HLA-matched tumor cell lines [8]. In later studies, this “selected” TIL strategy was exchanged for minimally cultured “young” TILs with an initial outgrowth phase < 20 days. During “young” TIL preparation, no pre-selection on tumor reactivity is used. All TILs that are grown are used for REP, making it easier to adapt [8, 49]. Interestingly, clinical response rates with “young” TILs are comparable with “selected” TIL [35, 50], which makes “young” TIL the current standard in the field.

Besides the ease, young TILs have two other major advantages; firstly, culture time is kept to a minimum. This is important since short culture times are associated with a better clinical response to TIL therapy [35]. Secondly, this optimization step results in a higher success rate to generate a clinical product, since for some patients no autologous tumor material or matching cell line is available, or no IFN-γ production could be observed.

### TIL selection

TIL products are heterogeneous products. Not only do they differ in percentage CD8^+^ versus CD4^+^ T cells, but also in tumor reactivity and antigen specificity. As discussed above, only a fraction (up to 30% in our hands) of the total population is tumor reactive. In order to enhance tumor reactivity, TIL could be enriched using a selection marker. Selecting for a tumor reactive population beforehand could ideally also shorten culture time and lower the number of infused cells.

In 2010 Rosenberg and colleagues showed that PD-1 expression is high on melanoma reactive TIL and that this marker could be used to pre-select tumor reactive cells from the bulk TIL population using FACS or magnetic bead sorting. After enrichment, the PD-1 positive T cells were expanded in standard REP protocol. Using this PD-1 selection method, in three out of five tested patients, TIL products showed enhanced tumor reactivity compared to the PD-1 negative or non-selected population [51].

In another study, Powell et al. showed that CD137/4-1BB, an activation marker for CD8^+^ T cells, could be used to select tumor reactive TILs from melanoma samples. TILs were either FACS sorted or bead selected based on CD137 expression, and also these selected cells showed enhanced tumor reactivity compared to unselected TIL. Both showed enhanced in vitro recognition of melanoma cells lines, based on IFN-γ production, and in vivo tumor control in a patient derived xenograft (PDX) mouse model was demonstrated [52]. Recently the Sheba Medical Center, Israel, demonstrated that CD137 selection could be performed with clinical grade compliant reagents. They expanded CD137 selected TILs in a large-scale manner to meet cell numbers required for patient treatment in a GMP facility. The increased anti-tumor effect was most prominent in an in vitro killing assay (using LDH release) and less prominent in IFN-γ release. Using this protocol, CD137 selected TILs were enriched for the recognition of both neo-antigens and shared antigens [45]. The Sheba Medical Center is currently running a trial using this CD137 selection strategy. Whether CD137 or PD-1 is the best marker to enrich for melanoma-reactive TIL is not known at present. Both methods will be further evaluated in clinical trials.

Our own group showed that the tumor reactivity of TIL products can be enhanced using clinical grade MHC streptamers to enrich for sub-populations of TIL with defined specificities. This strategy works for selection of TIL with both shared and neo-antigen reactivity. Importantly, the protocol can be performed under GMP conditions. A major challenge for clinical implementation of this strategy, is the requirement for knowledge of the peptide-specificity within the TIL product, before the MHC streptamers can be generated [53]. In addition, streptamers are only available for a limited number of HLA- alleles.

Several groups showed that infusion of high numbers of CD8^+^ TIL is associated with a higher objective response [17, 35]. Both total number and percentage of CD8^+^ cells is significantly correlated with objective response (*p* = 0.0003 and *p* = 0.001 respectively) [17]. In addition, the observation was made that the presence of CD4^+^FoxP3^+^ Tregs is associated with lower clinical activity of TILs [54], suggesting that CD4^+^ cells in the infusion product might negatively influence clinical activity. This hypothesis was tested in a randomized controlled trial (RCT) with TIL in melanoma patients in which CD8^+^ enriched and unselected “young” TIL were compared. This study failed to show higher clinical activity of the CD8^+^ selected TILs [46].

### Genetic editing of TIL

Current rapid developments in gene editing could also further enhance TIL therapy. These developments make it technically feasible to introduce potential beneficial receptors or molecules, or the other way around, knock-down/knock-out the ones that might be reducing the effect of TIL. Rosenberg and colleagues showed that Zinc Finger nuclease can be used to down regulate PD-1 in TIL, resulting in clinical grade TIL products with an enhanced effector function and cytokine production [55]. The currently widely used CRISPR-cas9 technology is expected to further increase the possibilities for gene editing of TIL. The MD Anderson Comprehensive Cancer Center, Houston, Texas, US, uses a lentiviral vector to transduce TIL with the chemokine receptor CXCR2, which could potentially improve tumor homing [56]. This strategy is currently evaluated in the clinic (see Table 1, NCT01740557). Transient, non-viral gene delivery by mRNA could also be used as alternative for the introduction of additional chemokine receptors in TIL [57]. All these technical developments open endless potential genetic improvements of the cell products.

**Fig. 1:**
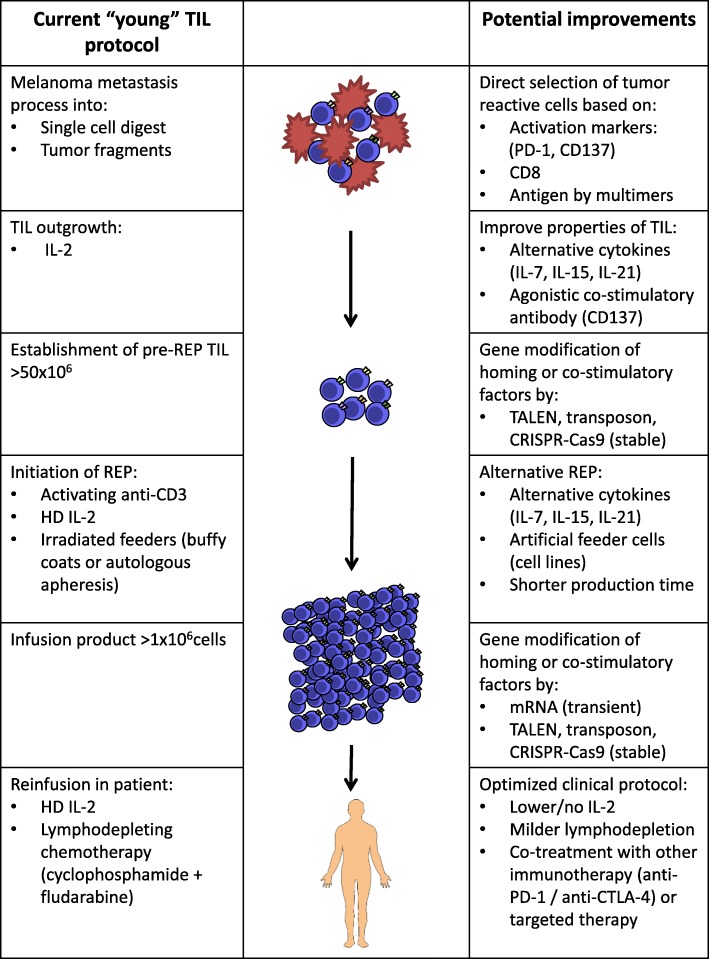
Schematic Overview of the Current TIL Production Protocol and Potential Improvements. Currently, surgically removed melanoma metastases are processed into single cell digest or smaller tumor pieces. At this point in production, direct selection of tumor reactive cells based on activation markers such as PD-1 or CD137, or CD8^+^ T cells or multimers can be applied. TIL outgrowth currently occurs in HD IL-2. Outgrowth of TIL could be improved in the presence of alternative cytokines such as IL-7, IL-15 or IL-21 or agonistic co-stimulatory antibodies such as CD137. In addition, a variation of gene modifications of homing or co-stimulatory factors can be applied. The current REP protocol consists of addition of activating soluble anti-CD3, HD IL-2 and irradiated feeders, but may be improved by addition of alternative cytokines such as IL-7, IL-15 and IL-21 and artificial feeders may be used. Also, the current REP time may be shortened. After REP, gene modification can also be applied. The infusion procedure of TIL to the patient currently consists of a conditioning lymphodepleting regimen, usually cyclophosphamide and fludarabine and administration of HD IL-2 following TIL infusion. However, multiple studies are being conducted with adjusted doses and treatment schedules of the lymphodepleting regimen and IL-2, as are studies being conducted with TIL as combination therapy to further potentiate the anti-tumor effect of TIL

An overview of the current TIL production protocol and potential improvements is shown in Fig. 1.

## TIL beyond monotherapy in stage IV melanoma

So far, response rates to TIL treatment have been consistent between the many small or larger phase I/II clinical trials [8, 9]. In order to improve overall response and survival, TIL therapy could be combined with other immunotherapies [58]. In addition, TIL treatment for other malignancies than melanoma has become feasible as well, creating new treatment possibilities for these patients.

### TIL as adjuvant therapy

Few studies with TIL treatment have been performed in patients with stage III melanoma. In a RCT conducted by Dreno et al., Nantes, France, 88 patients with stage III melanoma were treated with adjuvant TIL/IL-2 (*n* = 44) or IL-2 alone (*n* = 44) after surgery. Their hypothesis was that TIL treatment could be more efficacious in a setting with a minimal tumor burden. Patients receiving two infusions of 0.22–3.34 × 10^10^ TIL at 6 and 10 weeks post-surgery followed by daily s.c. IL-2 injection (6 × 10^6^ IU/m^2^) for 5 days a week for 2 weeks with each TIL infusion, showed superior relapse free survival (RFS) and OS compared to the s.c. IL-2 only [10, 59, 60]. Importantly, TIL infusions were not preceded by NMA lymphodepletion and the number of cells infused were ~ 10-fold lower compared to ‘classical’ TIL. As s.c. IL-2 is not approved as adjuvant therapy for patients with stage III melanoma, it is difficult to put the outcome of this study in perspective.

### Combination therapy with TIL

Recently, results have been published from a study in metastatic melanoma patients, who were treated with the combination of a targeted agent plus TIL. In this pilot study in 11 patients with BRAF^V600E/K^ mutated melanoma, the BRAF inhibitor vemurafenib was given in conjunction with TIL. Patients were treated with vemurafenib for two weeks following metastectomy for the production of TIL, after which another lesion was resected. Patients were further treated according to standard protocol of lymphodepleting regimen, TIL infusion and IL-2. Vemurafenib was resumed after TIL infusion and continued for two years. Seven out of 11 patients (64%) showed an objective clinical response, two of whom had a durable response lasting up to three years [61]. These results are promising, however larger, randomized studies are needed to show the value of this approach in comparison to TIL alone. Currently, two clinical trials in which targeted therapy is being combined with TIL are actively accruing patients (NCT02354690, NCT01659151), see Table 1.

Treatment with the anti-CTLA-4 drug ipilimumab, was shown to heighten T cell infiltration into melanomas and to broaden the TIL response to these tumors [62]. In a recent clinical trial at the Moffit Cancer Center, Tampa, US, 13 patients with metastatic melanoma were treated with ipilimumab in combination with standard TIL therapy. Patients received four doses of ipilimumab (3 mg/kg), starting two weeks before metastectomy for TIL harvest, one week after resection of a metastasis, followed by two and five weeks after conditioning chemotherapy. Five out of 13 patients (38.5%) showed an OR, four of which were durable, lasting up to one year and one patient developed a CR 52 months after this treatment [11]. Response rates seen in this trial were not different from those in other TIL trials. However, these data are the first to demonstrate the feasibility if combining TIL with immune checkpoint blockade.

Currently, several trials have been initiated combining TIL with PD-1 blocking agents (NCT03374839, NCT03475134, NCT03158935, NCT02652455, NCT02621021, NCT01993719), see also Table 1. Synergism from this combination may be expected as the ex vivo grown and expanded tumor-reactive TIL are often PD-1 positive [63] and prevention of the interaction between PD-1 on T cells and PDL-1 on tumor cells by anti-PD-1 therapy around the time of TIL infusion, may render these TIL more tumoricidal.

In addition, other immunotherapy modalities such as dendritic cell vaccination and (peg-)interferon, are being evaluated in a clinical setting combined with TIL therapy. See also Table 1 for details on current recruiting trials of combinations with TIL.

### TIL therapy for other solid tumor types

For decades TIL treatment has been studied in patients with mostly metastatic cutaneous melanoma. Recently, investigators were also successful in growing out tumor reactive TILs from other tumor types, such as renal cell, breast and cervical cancer. In general, the tumor reactivity of TILs from these other tumors is lower when compared to melanoma [64]. The production and reactivity of TIL products for these other solid tumor types varies, amongst others, due to the heterogeneity in mutational load, and thus neo-antigens, and lymphocytic infiltration with variations of CD4^+^ and CD8^+^ T cells [65].

Promising ORR of up to 35% have been seen in patients with metastatic uveal melanoma in an ongoing single-center, single-arm, phase II TIL study with 21 patients [66]. Despite the for this disease impressive ORR, the durability of these responses appeared short compared to what has been observed for cutaneous melanoma. A phase II trial has opened to confirm these results in a larger cohort, NCT03467516, see Table 1.

Recently, successful isolation, expansion and tumor recognition of TIL from renal cell carcinoma was reported. However, the reactivity of TIL was weaker and showed reduced functionality compared to TIL from melanomas [67]. Also in breast cancer, it is possible to isolate and expand TIL ex vivo under standard culture conditions. Four out of six randomly selected post-REP TIL samples were found to be reactive to the autologous tumor in vitro, which also showed functionality in vivo in a xenograft mouse model [12]. Recently, Stevanovic et al. demonstrated clinical responses upon TIL treatment in patients with refractory metastatic cervical cancer, with three of the nine treated patients showing objective tumor regression, two of which were durable. When possible, TILs were selected for HPV E6 and E7 reactivity, as the vast majority of cervical cancers harbor HPV oncoproteins that may act as immunotherapeutic targets for TIL [13]. Currently, a “basket” clinical phase II study is being conducted at the NIH in patients with a variety of metastatic disease, including digestive tract, breast, urothelial, ovarian and endometrial cancers, in order to provide information about rates of tumor regression when treated with TIL (NCT01174121).

## Limitations of TIL therapy and conclusions

Despite the many promising beneficial effects, TIL therapy clearly also has its limitations. Firstly, TIL is the ultimate personalized immunotherapy, as for every individual patient a specific infusion product needs to be produced. Since infusion products have to be produced in a patient specific manner, costs are relatively high. However, non-commercial prices for TIL treatment are still considerably lower compared to treatment with checkpoint inhibitors, such as anti-CTLA-4, as described in an early cost-effectiveness model for TIL versus ipilimumab in patients with metastatic melanoma [68]. As discussed, success rates of TIL outgrowth vary between 75 and 97% [8, 18]. Therefore, there is a risk for every patient that treatment needs to be canceled. Production time of a TIL product is more than one month, which may be too long for some patients with rapidly progressive disease. In addition, highly specialized GMP facilities and production staff need to be in place, which requires extensive investments and training. The development of computerized bioreactors could, at least in part, take over some of the handling by production staff in the near future, although the heterogeneity of the original material (tumor fragments or digest), makes it difficult to use a fully automated production process.

Overall, treatment with TIL shows great possibilities as anti-cancer therapy in melanoma and in the future, possibly also in other solid tumors. However, TIL has not been approved as anti-cancer treatment yet by the regulatory authorities due to lack of results coming from sufficiently powered prospective RCTs. The currently recruiting phase III trial as discussed above (NCT02278887) should give the first direct proof of the effectiveness of TIL treatment compared to the current standard of care in patients with advanced melanoma unresponsive upon prior treatment.

## Additional file

Additional file 1: Additional file 1: **Table S1.** Completed and Published Trials with Tumor-infiltrating Lymphocytes in Patients with Melanoma. (DOCX 58 kb)

## Abbreviations

ACT: Adoptive cell therapy
AE: Adverse event
b.i.d.: Bis in die
C/T: Cancer/testis
CCIT: Center for Cancer Immune Therapy
CD: Cluster of differentiation
CDK4: Cyclin-dependent kinase 4
CR: Complete remission
CTLA-4: Cytotoxic T-lymphocyte-associated protein-4
CXCR: C-X-C chemokine receptor
Cy: Cyclophosphamide
d: Day
DC: Dendritic cell
DNA: Deoxyribonucleic acid
FACS: Fluorescence-activated cell sorting
Flu: Fludarabine
FoxP3: Forkhead box P
GMP: Good manufacturing practice
Gp100: Glycoprotein 100
Gy: Gray
HD: High-dose
HLA: Human leukocyte antigen
HPV: Human papillomavirus
h: Hour
i.d: Intradermal
i.v.: Intravenous
IFN: Interferon
IL: Interleukin
Ipi: Ipilimumab
IU: International unit
kg: Kilogram
LD: Low-dose
LDH: Lactate dehydrogenase
LN-144: TIL production technology developed by Iovance Biotherapeutics
LPS: Lipopolysaccharide
MART-1: Melanoma antigen recognized by T cells 1
max: Maximum
MD: Melanoma differentiation
mg: Milligram
MHC: Major histocompatibility complex
MIU: Million international units
mRNA: Messenger RNA
NA: Not available
NGFR: Nerve growth factor receptor
NHS: National Health Service
NIH: National Institutes of Health
Nivo: Nivolumab
NKI: Netherlands Cancer Institute
NMA: Non-myeloablative
OE: Overexpressed
ORR: Objective response rate
OS: Overall survival
PD: Progressive disease
PD-1: Programmed death protein-1
PDL-1: Programmed death ligand-1
PDX: Patient derived xenograft
Pembro: Pembrolizumab
PFS: Progression free survival
PR: Partial response
q: Every
RCT: Randomized controlled trial
REP: Rapid Expansion Protocol
RFS: Relapse free survival
s.c.: Subcutaneous
t.i.d.: Ter in die
TBI: Total body irradiation
TCR: T cell receptor
TIL: Tumor-infiltrating lymphocytes
TNF: Tumor necrosis factor
Treg: Regulatory T cell
Vem: Vemurafenib
w: Week
x: Times
yr: Year

## References

[1] Karimkhani C, Green AC, Nijsten T (2017). The global burden of melanoma: results from the global burden of disease study 2015. Br J Dermatol.

[2] Luke JJ, Flaherty KT, Ribas A, Long GV (2017). Targeted agents and immunotherapies: optimizing outcomes in melanoma. Nat Rev Clin Oncol.

[3] Svedman FC, Pillas D, Taylor A (2016). Stage-specific survival and recurrence in patients with cutaneous malignant melanoma in Europe - a systematic review of the literature. Clin Epidemiol.

[4] Wolchok JD, Chiarion-Sileni V, Gonzalez R (2017). Overall survival with combined Nivolumab and Ipilimumab in advanced melanoma. N Engl J Med.

[5] June CH, Riddell SR, Schumacher TN (2015). Adoptive cellular therapy: a race to the finish line. Sci Transl Med.

[6] Rosenberg SA, Yannelli JR, Yang JC (1994). Treatment of patients with metastatic melanoma with autologous tumor-infiltrating lymphocytes and interleukin 2. J Natl Cancer Inst.

[7] Dudley ME, Wunderlich JR, Robbins PF (2002). Cancer regression and autoimmunity in patients after clonal repopulation with antitumor lymphocytes. Science.

[8] Rosenberg SA, Yang JC, Sherry RM (2011). Durable complete responses in heavily pretreated patients with metastatic melanoma using T-cell transfer immunotherapy. Clin Cancer Res.

[9] Wu R, Forget MA, Chacon J (2012). Adoptive T-cell therapy using autologous tumor-infiltrating lymphocytes for metastatic melanoma: current status and future outlook. Cancer J.

[10] Khammari A, Knol AC, Nguyen JM (2014). Adoptive TIL transfer in the adjuvant setting for melanoma: long-term patient survival. J Immunol Res.

[11] Mullinax JE, Hall M, Prabhakaran S (2018). Combination of Ipilimumab and adoptive cell therapy with tumor-infiltrating lymphocytes for patients with metastatic melanoma. Front Oncol.

[12] Lee HJ, Kim YA, Sim CK (2017). Expansion of tumor-infiltrating lymphocytes and their potential for application as adoptive cell transfer therapy in human breast cancer. Oncotarget.

[13] Stevanovic S, Draper LM, Langhan MM (2015). Complete regression of metastatic cervical cancer after treatment with human papillomavirus-targeted tumor-infiltrating T cells. J Clin Oncol.

[14] Markel G, Cohen-Sinai T, Besser MJ (2009). Preclinical evaluation of adoptive cell therapy for patients with metastatic renal cell carcinoma. Anticancer Res.

[15] Besser MJ, Shapira-Frommer R, Itzhaki O (2013). Adoptive transfer of tumor-infiltrating lymphocytes in patients with metastatic melanoma: intent-to-treat analysis and efficacy after failure to prior immunotherapies. Clin Cancer Res.

[16] Dudley ME, Yang JC, Sherry R (2008). Adoptive cell therapy for patients with metastatic melanoma: evaluation of intensive myeloablative chemoradiation preparative regimens. J Clin Oncol.

[17] Radvanyi LG, Bernatchez C, Zhang M (2012). Specific lymphocyte subsets predict response to adoptive cell therapy using expanded autologous tumor-infiltrating lymphocytes in metastatic melanoma patients. Clin Cancer Res.

[18] Andersen R, Donia M, Ellebaek E (2016). Long-lasting complete responses in patients with metastatic melanoma after adoptive cell therapy with tumor-infiltrating lymphocytes and an attenuated IL2 regimen. Clin Cancer Res.

[19] Dudley ME, Wunderlich JR, Yang JC (2005). Adoptive cell transfer therapy following non-myeloablative but lymphodepleting chemotherapy for the treatment of patients with refractory metastatic melanoma. J Clin Oncol.

[20] Rosenberg SA, Spiess P, Lafreniere R (1986). A new approach to the adoptive immunotherapy of cancer with tumor-infiltrating lymphocytes. Science.

[21] Mills CD, North RJ (1983). Expression of passively transferred immunity against an established tumor depends on generation of cytolytic T cells in recipient. Inhibition by suppressor T cells. J Exp Med.

[22] Antony PA, Piccirillo CA, Akpinarli A (2005). CD8+ T cell immunity against a tumor/self-antigen is augmented by CD4+ T helper cells and hindered by naturally occurring T regulatory cells. J Immunol.

[23] Gattinoni L, Finkelstein SE, Klebanoff CA (2005). Removal of homeostatic cytokine sinks by lymphodepletion enhances the efficacy of adoptively transferred tumor-specific CD8+ T cells. J Exp Med.

[24] Schluns KS, Kieper WC, Jameson SC, Lefrancois L (2000). Interleukin-7 mediates the homeostasis of naive and memory CD8 T cells in vivo. Nat Immunol.

[25] Schluns KS, Williams K, Ma A (2002). Cutting edge: requirement for IL-15 in the generation of primary and memory antigen-specific CD8 T cells. J Immunol.

[26] Goff SL, Dudley ME, Citrin DE (2016). Randomized, prospective evaluation comparing intensity of Lymphodepletion before adoptive transfer of tumor-infiltrating lymphocytes for patients with metastatic melanoma. J Clin Oncol.

[27] McDermott DF, Atkins MB (2006). Interleukin-2 therapy of metastatic renal cell carcinoma--predictors of response. Semin Oncol.

[28] Marabondo S, Kaufman HL (2017). High-dose interleukin-2 (IL-2) for the treatment of melanoma: safety considerations and future directions. Expert Opin Drug Saf.

[29] Malek TR (2008). The biology of interleukin-2. Annu Rev Immunol.

[30] Dudley ME, Wunderlich JR, Yang JC (2002). A phase I study of nonmyeloablative chemotherapy and adoptive transfer of autologous tumor antigen-specific T lymphocytes in patients with metastatic melanoma. J Immunother.

[31] Ellebaek E, Iversen TZ, Junker N (2012). Adoptive cell therapy with autologous tumor infiltrating lymphocytes and low-dose Interleukin-2 in metastatic melanoma patients. J Transl Med.

[32] Yang JC (2015). Toxicities associated with adoptive T-cell transfer for Cancer. Cancer J.

[33] Johnson LA, Morgan RA, Dudley ME (2009). Gene therapy with human and mouse T-cell receptors mediates cancer regression and targets normal tissues expressing cognate antigen. Blood.

[34] Yeh S, Karne NK, Kerkar SP (2009). Ocular and systemic autoimmunity after successful tumor-infiltrating lymphocyte immunotherapy for recurrent, metastatic melanoma. Ophthalmol.

[35] Itzhaki O, Hovav E, Ziporen Y (2011). Establishment and large-scale expansion of minimally cultured “young” tumor infiltrating lymphocytes for adoptive transfer therapy. J Immunother.

[36] Kvistborg P, Shu CJ, Heemskerk B (2012). TIL therapy broadens the tumor-reactive CD8(+) T cell compartment in melanoma patients. Oncoimmunology.

[37] Wolfel T, Hauer M, Schneider J (1995). A p16INK4a-insensitive CDK4 mutant targeted by cytolytic T lymphocytes in a human melanoma. Science.

[38] Matsushita H, Vesely MD, Koboldt DC (2012). Cancer exome analysis reveals a T-cell-dependent mechanism of cancer immunoediting. Nature.

[39] Castle JC, Kreiter S, Diekmann J (2012). Exploiting the mutanome for tumor vaccination. Cancer Res.

[40] Rizvi NA, Hellmann MD, Snyder A (2015). Cancer immunology. Mutational landscape determines sensitivity to PD-1 blockade in non-small cell lung cancer. Science.

[41] Snyder A, Makarov V, Merghoub T (2014). Genetic basis for clinical response to CTLA-4 blockade in melanoma. N Engl J Med.

[42] Le DT, Uram JN, Wang H (2015). PD-1 blockade in tumors with mismatch-repair deficiency. N Engl J Med.

[43] Le DT, Durham JN, Smith KN (2017). Mismatch repair deficiency predicts response of solid tumors to PD-1 blockade. Science.

[44] Tran E, Turcotte S, Gros A (2014). Cancer immunotherapy based on mutation-specific CD4+ T cells in a patient with epithelial cancer. Science.

[45] Seliktar-Ofir S, Merhavi-Shoham E, Itzhaki O (2017). Selection of shared and Neoantigen-reactive T cells for adoptive cell therapy based on CD137 separation. Front Immunol.

[46] Dudley ME, Gross CA, Somerville RP (2013). Randomized selection design trial evaluating CD8+−enriched versus unselected tumor-infiltrating lymphocytes for adoptive cell therapy for patients with melanoma. J Clin Oncol.

[47] Donia M, Larsen SM, Met O, Svane IM (2014). Simplified protocol for clinical-grade tumor-infiltrating lymphocyte manufacturing with use of the Wave bioreactor. Cytotherapy.

[48] Kaneko S, Mastaglio S, Bondanza A (2009). IL-7 and IL-15 allow the generation of suicide gene-modified alloreactive self-renewing central memory human T lymphocytes. Blood.

[49] Tran KQ, Zhou J, Durflinger KH (2008). Minimally cultured tumor-infiltrating lymphocytes display optimal characteristics for adoptive cell therapy. J Immunother.

[50] Dudley ME, Gross CA, Langhan MM (2010). CD8+ enriched “young” tumor infiltrating lymphocytes can mediate regression of metastatic melanoma. Clin Cancer Res.

[51] Inozume T, Hanada K, Wang QJ (2010). Selection of CD8+PD-1+ lymphocytes in fresh human melanomas enriches for tumor-reactive T cells. J Immunother.

[52] Ye Q, Song DG, Poussin M (2014). CD137 accurately identifies and enriches for naturally occurring tumor-reactive T cells in tumor. Clin Cancer Res.

[53] Kelderman S, Heemskerk B, Fanchi L (2016). Antigen-specific TIL therapy for melanoma: a flexible platform for personalized cancer immunotherapy. Eur J Immunol.

[54] Yao X, Ahmadzadeh M, Lu YC (2012). Levels of peripheral CD4(+)FoxP3(+) regulatory T cells are negatively associated with clinical response to adoptive immunotherapy of human cancer. Blood.

[55] Beane JD, Lee G, Zheng Z (2015). Clinical scale zinc finger nuclease-mediated gene editing of PD-1 in tumor infiltrating lymphocytes for the treatment of metastatic melanoma. Mol Ther.

[56] Forget MA, Tavera RJ, Haymaker C (2017). A novel method to generate and expand clinical-grade, genetically modified, Tumor-Infiltrating Lymphocytes. Front Immunol.

[57] Idorn M, Thor Straten P, Svane IM, Met O (2016). Transfection of tumor-infiltrating T cells with mRNA encoding CXCR2. Methods Mol Biol.

[58] Foley KC, Nishimura MI, Moore TV (2018). Combination immunotherapies implementing adoptive T-cell transfer for advanced-stage melanoma. Melanoma Res.

[59] Dreno B, Nguyen JM, Khammari A (2002). Randomized trial of adoptive transfer of melanoma tumor-infiltrating lymphocytes as adjuvant therapy for stage III melanoma. Cancer Immunol Immunother.

[60] Khammari A, Nguyen JM, Pandolfino MC (2007). Long-term follow-up of patients treated by adoptive transfer of melanoma tumor-infiltrating lymphocytes as adjuvant therapy for stage III melanoma. Cancer Immunol Immunother.

[61] Deniger DC, Kwong ML, Pasetto A (2017). A pilot trial of the combination of Vemurafenib with adoptive cell therapy in patients with metastatic melanoma. Clin Cancer Res.

[62] Tarhini AA, Edington H, Butterfield LH (2014). Immune monitoring of the circulation and the tumor microenvironment in patients with regionally advanced melanoma receiving neoadjuvant ipilimumab. PLoS One.

[63] Donia M, Kjeldsen JW, Andersen R (2017). PD-1(+) Polyfunctional T cells dominate the periphery after tumor-infiltrating lymphocyte therapy for Cancer. Clin Cancer Res.

[64] Yannelli JR, Hyatt C, McConnell S (1996). Growth of tumor-infiltrating lymphocytes from human solid cancers: summary of a 5-year experience. Int J Cancer.

[65] Radvanyi LG (2015). Tumor-infiltrating lymphocyte therapy: addressing prevailing questions. Cancer J.

[66] Chandran SS, Somerville RPT, Yang JC (2017). Treatment of metastatic uveal melanoma with adoptive transfer of tumour-infiltrating lymphocytes: a single-Centre, two-stage, single-arm, phase 2 study. Lancet Oncol.

[67] Andersen R, Westergaard MCW, Kjeldsen JW (2018). T-cell responses in the microenvironment of primary renal cell carcinoma-implications for adoptive cell therapy. Cancer Immunol Res.

[68] Retel VP, Steuten LM, Mewes JC, van Harten WH (2014). Early cost-effectiveness modeling for tumor infiltrating lymphocytes (TIL) -treatment versus Ipilimumab in metastatic melanoma patients. Value Health.

