# Supplementary material for: Adoptive transfer of tumor-infiltrating lymphocytes in melanoma: a viable treatment option
Source: J Immunother Cancer. 2018 Oct 3;6:102. doi: 10.1186/s40425-018-0391-1 (PMC6171186; doi:10.1186/s40425-018-0391-1)
Supplement: Supplementary file 1 — Additional file 1: Table S1. Completed and Published Trials with Tumor-infiltrating Lymphocytes in Patients with Melanoma. (DOCX 58 kb) [file 40425_2018_391_MOESM1_ESM.docx]

**Additional Table 1. Completed and Published Trials with Tumor-infiltrating Lymphocytes in Patients with Melanoma**

| **Group** | **Phase** | **Patients enrolled** | **Disease stage**  **melanoma** | **Intervention** | **TIL product and number of cells infused (range or mean ± SEM)** | **Preparative lymphodepleting regimen** | **IL-2 regimen** | **Response (OR) (%) according to RECIST of treated patients (n=)** |
| --- | --- | --- | --- | --- | --- | --- | --- | --- |
| **Mullinax et al., 2018 [1]**  **Moffit Cancer Center, Tampa, US** | II | 13 | III/IV | Ipi (4 doses 3mg/kg) 2w prior to metastectomy, 1w post metastectomy, 2 and 5 w post lymphodepletion) + TIL + HD IL-2 | Young TIL  2.3×10^10^ – 1.0×10^11^ | Cy 60 mg/kg for 2 d + Flu 25 mg/m^2^ for 5 d | 720,000 IU/kg t.i.d. until tolerable toxicity , max 15 doses | 38.5 (n=13) |
| **Chandran et al., 2017 [2]**  **NIH, Bethesda, Maryland, US** | II | 21 | Metastatic ocular melanoma | Cohort 1: Lymphodepletion + TIL + HD IL-2  Cohort 2: Lymphodepletion + TIL | Cohort 1+2: young TIL 1x10^9^ – 2x10^11^ | Cohort 1+ 2: Cy 60 mg/kg for 2 d + Flu 25 mg/m^2^ for 5 d | Cohort 1: 720,000 IU/kg t.i.d. until tolerable toxicity, max 15 doses  Cohort 2: No IL-2 | 35 (n=21) |
| **Andersen et al., 2016 [3]**  **CCIT, Herlev, Denmark** | I/II | 25 | IIIc/IV | Lymphodepletion + TIL + LD IL-2 | Young TIL  10^9^-10^10^ | Cy 60 mg/kg for 2 d + Flu 25 mg/ m^2^ for 5 d | Decrescendo regimen (18 MIU/m^2^ for 6 hr, 18 MIU/ m^2^ for 12 hr, 18 MIU/ m^2^ for 24 hr followed by 4.5 MIU/ m^2^ for another 3 x 24 hr) | 42 (n=25) |
| **Khammari et al., 2014 [4]**  **Nantes University Hospital, Nantes, France** | III | 88 | III | Cohort 1: Adjuvant TIL + LD IL-2  Cohort 2: Adjuvant LD IL-2 | TIL injection at 6 + 10 w post-surgery  0.22 – 3.34x10^10^ | - | s.c. injection (6x10^6^ IU/m^2^) 5 d per w for 2 w | RFS cohort 1: 66 (n=44)  RFS cohort 2: 70 (n=44) |
| **Besser et al., 2013 [5]**  **Sheba Medical Center, Israel** | II | 80 | IV | Lymphodepletion + TIL + HD IL-2 | Young TIL  52 ± 24x10^9^ | Cy 60 mg/kg for 2 d + Flu 25 mg/ m^2^ for 5 d | 720,000 IU/kg t.i.d. until tolerable toxicity, max 15 doses | 40 (n=57) |
| **Dudley et al., 2013 [6]**  **NIH, Bethesda, Maryland, US** | II | 101 | IV | Cohort 1+2: Lymphodepletion + TIL + HD IL-2 | Cohort 1: Unselected young TIL 9.8 – 84.9x10^9^  Cohort 2: CD8^+^ young TIL 4.5 – 147.0x10^9^ | Cy 60 mg/kg for 2 d + Flu 25 mg/ m^2^ for 5 d | 720,000 IU/kg t.i.d. until tolerable toxicity, max 15 doses | Cohort 1: 35 (n=34)  Cohort 2: 20 (n=35)  Total: 29 (n=69) |
| **Radvanyi et al., 2012 [7]**  **MD Anderson Cancer Center, Houston, Texas, US** | II | 31 | IIIc/IV | Lymphodepletion + TIL + 2 cycles HD IL-2 | Young TIL  (8 – 150 × 10^9^) | Cy 60 mg/kg for 2 d + Flu 25 mg/ m^2^ for 5 d | 1^st^ cycle: 720,000 IU/kg t.i.d. until tolerable toxicity, max 15 doses  2^nd^ cycle: 21d post TIL | 42 (n=31) |
| **Pilon-Thomas et al., 2012 [8]**  **Moffitt Cancer Center and Research Institute, Tampa, Florida, US** | Pilot | 19 | III/IV | Lymphodepletion + TIL + HD IL-2 | Selected TIL  (2.0 – 11.0x10^11^) | Cy 60 mg/kg for 2 d + Flu 25 mg/ m^2^ for 5 d | 720,000 IU/kg t.i.d. until tolerable toxicity, max 15 doses | 38 (n=13) |
| **Ullenhag et al., 2012 [9]**  **Uppsala University, Uppsala, Sweden** | II | 24 | IV | Lymphodepletion + TIL + LD IL-2 | Selected TIL  (0.5 – 30.0x10^9^) | Cy 60 mg/kg for 2 d + Flu 25 mg/ m^2^ for 5 d | s.c. injection (2.4x10^6^ units/m^2^) until PD or unacceptable toxicity | 21 (n=24) |
| **Rosenberg et al., 2011 [10]**  **NIH, Bethesda, Maryland, US** | II | 93 | IV | Lymphodepletion + TIL + HD IL-2 | Selected TIL  6.5 ± 0.7x10^10^ (CR n= 20)  6.1 ± 0.5x10^10^ (PR n=32)  5.5 ± 0.6 x10^10^(NR n=41) | Cohort 1: Cy 60 mg/kg for 2 d + Flu 25 mg/ m^2^ for 5 d  Cohort 2: Cy 60 mg/kg for 2 d + Flu 25 mg/ m^2^ for 5 d + TBI 2 Gy  Cohort 3: Cy 60 mg/kg for 2 d + Flu 25 mg/ m^2^ for 5 d + TBI 2 x 2 Gy/d for 3 d | 720,000 IU/kg t.i.d. until tolerable toxicity, max 15 doses | Cohort 1: 49 (n=43)  Cohort 2: 52 (n=25)  Cohort 3: 72 (n=25)  Total: 56 (n=93) |
| **Itzhaki et al., 2011 [11]**  **Sheba Medical Center, Israel** | II | 55 | IV | Lymphodepletion + TIL + HD IL-2 | Young TIL  4.5 ± 2.0x10^10^ | Cy 60 mg/kg for 2 d + Flu 25 mg/ m^2^ for 5 d | 720,000 IU/kg t.i.d. until tolerable toxicity, max 15 doses | 48 (n=31) |
| **Dudley et al., 2010 [12]**  **NIH, Bethesda, Maryland, US** | II | 122 | IV | Cohort 1 + 2: Lymphodepletion + TIL + HD IL-2 | CD8^+^ enriched young TIL  Cohort 1: 47.7 ± 3.3 x10^9^  Cohort 2: 43.1 ±7.5 x10^9^ | Cohort 1: Cy 60 mg/kg for 2 d + Flu 25 mg/ m^2^ for 5 d  Cohort 2: Cy 60 mg/kg for 2 d + Flu 25 mg/ m^2^ for 5 d + TBI 3 x 2Gy | 720,000 IU/kg t.i.d. until tolerable toxicity, max 15 doses | Cohort 1: 58 (n=33)  Cohort 2: 48 (n=23)  Total: 54 (n=56) |
| **Dudley et al., 2002 [13]**  **NIH, Bethesda, Maryland, US** | I | 15 | IV | Cohort 1+2: Lymphodepletion + TIL  Cohort 3: Lymphodepletion + TIL + IL-2  Cohort 4: Lymphodepletion + TIL + IL-2  11 patients were treated with a 2^nd^ cycle | Selected TIL  (0.9 – 24.2x10^9^) | Cohort 1: Cy 30 mg/kg for 2 d + Flu 25 mg/ m^2^ for 5 d  Cohort 2-4: Cy 60 mg/kg for 2 d + Flu 25 mg/ m^2^ for 5 d | Cohort 3: 72,000 IU/kg t.i.d. for 5d  Cohort 4: 720,000 IU/kg t.i.d. until tolerable toxicity, max 12 doses | Cohort 1: 0 (n=3)  Cohort 2: 0 (n=3)  Cohort 3: 0 (n=3)  Cohort 4: 0 (n=6)  Total: 0 (n=15)  5 (30%) patients showed MR/transient regression of tumor |
| **Schwartzentruber et al., 1994 [14]**  **NIH, Bethesda, Maryland, US** | I/II | 43  (2 patients received >1 treatment) | IV | TIL + IL-2 with or without prior lymphodepletion | Selected TIL  1.9 ± 0.1x10^10^ (OR n=9)  1.5 ± 0.1x10^10^ (NR n=34) | 16 patients received Cy 25mg/kg single infusion | 720,000 IU/kg t.i.d. until tolerable toxicity or 216,000 IU/kg + IFN-α 3x10^6^ U/m^2^ t.i.d. until tolerable toxicity | 21 (n=43) |
| **Rosenberg et al., 1994 [15]**  **NIH, Bethesda, Maryland, US** | I/II | 86 | IV | TIL + IL-2 in 2 cycles with or without prior lymphodepletion | Selected TIL  >10^11^ | 57 patients received Cy 25 mg/kg single infusion | 720,000 IU/kg t.i.d., max 15 doses  Repeated after 21 days | 34 (n=86) |

**Abbreviations:** CCIT, Center for Cancer Immune Therapy; CR, complete response; Cy, cyclophosphamide; d, day; Flu, fludarabine; Gy, Gray; HD, high-dose; hr, hour; i.v., intravenous; IFN-α, interferon alpha; IL-2, interleukin-2; Ipi, ipilimumab; IU, international unit; kg, kilogram; LD, low-dose; max, maximum; mg, milligram; MIU, million international units; MR, mixed response; NIH, National Institutes of Health; NR, no response; OR, objective response; PD, progressive disease; PR, partial response; RECIST, response evaluation criteria in solid tumors; s.c., subcutaneous; SEM, standard error of mean; t.i.d., ter in die; TIL, tumor-infiltrating lymphocytes; US, United States; w, week.

**References Additional Table 1:**

1. Mullinax JE, Hall M, Prabhakaran S et al. Combination of Ipilimumab and Adoptive Cell Therapy with Tumor-Infiltrating Lymphocytes for Patients with Metastatic Melanoma. Front Oncol 2018; 8: 44.

2. Chandran SS, Somerville RPT, Yang JC et al. Treatment of metastatic uveal melanoma with adoptive transfer of tumour-infiltrating lymphocytes: a single-centre, two-stage, single-arm, phase 2 study. Lancet Oncol 2017; 18: 792-802.

3. Andersen R, Donia M, Ellebaek E et al. Long-Lasting Complete Responses in Patients with Metastatic Melanoma after Adoptive Cell Therapy with Tumor-Infiltrating Lymphocytes and an Attenuated IL2 Regimen. Clin Cancer Res 2016; 22: 3734-3745.

4. Khammari A, Knol AC, Nguyen JM et al. Adoptive TIL transfer in the adjuvant setting for melanoma: long-term patient survival. J Immunol Res 2014; 2014: 186212.

5. Besser MJ, Shapira-Frommer R, Itzhaki O et al. Adoptive Transfer of Tumor Infiltrating Lymphocytes in Metastatic Melanoma Patients: Intent-to-Treat Analysis and Efficacy after Failure to Prior Immunotherapies. Clin Cancer Res 2013.

6. Dudley ME, Gross CA, Somerville RP et al. Randomized selection design trial evaluating CD8+-enriched versus unselected tumor-infiltrating lymphocytes for adoptive cell therapy for patients with melanoma. J Clin Oncol 2013; 31: 2152-2159.

7. Radvanyi LG, Bernatchez C, Zhang M et al. Specific lymphocyte subsets predict response to adoptive cell therapy using expanded autologous tumor-infiltrating lymphocytes in metastatic melanoma patients. Clin Cancer Res 2012; 18: 6758-6770.

8. Pilon-Thomas S, Kuhn L, Ellwanger S et al. Efficacy of adoptive cell transfer of tumor-infiltrating lymphocytes after lymphopenia induction for metastatic melanoma. J Immunother 2012; 35: 615-620.

9. Ullenhag GJ, Sadeghi AM, Carlsson B et al. Adoptive T-cell therapy for malignant melanoma patients with TILs obtained by ultrasound-guided needle biopsy. Cancer Immunol Immunother 2012; 61: 725-732.

10. Rosenberg SA, Yang JC, Sherry RM et al. Durable complete responses in heavily pretreated patients with metastatic melanoma using T-cell transfer immunotherapy. Clin Cancer Res 2011; 17: 4550-4557.

11. Itzhaki O, Hovav E, Ziporen Y et al. Establishment and large-scale expansion of minimally cultured "young" tumor infiltrating lymphocytes for adoptive transfer therapy. J Immunother 2011; 34: 212-220.

12. Dudley ME, Gross CA, Langhan MM et al. CD8+ enriched "young" tumor infiltrating lymphocytes can mediate regression of metastatic melanoma. Clin Cancer Res 2010; 16: 6122-6131.

13. Dudley ME, Wunderlich JR, Yang JC et al. A phase I study of nonmyeloablative chemotherapy and adoptive transfer of autologous tumor antigen-specific T lymphocytes in patients with metastatic melanoma. J Immunother 2002; 25: 243-251.

14. Schwartzentruber DJ, Hom SS, Dadmarz R et al. In vitro predictors of therapeutic response in melanoma patients receiving tumor-infiltrating lymphocytes and interleukin-2. J Clin Oncol 1994; 12: 1475-1483.

15. Rosenberg SA, Yannelli JR, Yang JC et al. Treatment of patients with metastatic melanoma with autologous tumor-infiltrating lymphocytes and interleukin 2. J Natl Cancer Inst 1994; 86: 1159-1166.
